# Analgesic efficacy of nefopam for cancer pain: a randomized controlled study

**DOI:** 10.12688/f1000research.23455.1

**Published:** 2020-05-19

**Authors:** Koravee Pasutharnchat, Wichita Wichachai, Rungrawan Buachai

**Affiliations:** 1 Department of Anesthesiology, Faculty of Medicine, Ramathibodi Hospital, Mahidol University, Bangkok, 10400, Thailand

**Keywords:** cancer, pain, nefopam, analgesia, efficacy, patient controlled analgesia, morphine consumption, side effect, analgesia, pain reduction

## Abstract

**Background:** Nefopam is a non-opioid, non-steroidal, central acting drug used effectively for postoperative pain. The efficacy of nefopam for cancer pain remains unclear. We aimed to evaluate the analgesic efficacy of nefopam for cancer pain in a randomized controlled trial.

**Methods**: Patients with moderate to severe cancer pain (n=40) were randomly divided into two groups. The nefopam group (n=20) received three 20 mg doses of nefopam every 8 hours. The placebo group (n=20) received normal saline. Intravenous patient-controlled analgesia with morphine was given for breakthrough pain for 48 hours. The primary outcome was significant pain reduction. Secondary outcomes were morphine consumption over 48 hours and incidence of side effects.

**Results:** The nefopam group showed pain reduction at 12 hours (65% of patients), 24 hours (80%), 36 hours (85%), and 48 hours (65%). The placebo group showed pain reduction at 12 hours (70%), 24 hours (75%), 36 hours (80%), and 48 hours (60%). However, there were no statistically significant differences between the groups (p>0.05). The median dosage of morphine consumption in 48 hours was lower in the nefopam group (25.5 mg) compared with the placebo group (37 mg), but this was not statistically significant (p=0.499). There were no statistically significant differences in blood pressure and heart rate between the groups. Side effects in both groups were comparable.

**Conclusions:** At dosage of 60 mg in 24 hours, nefopam did not provide significant pain reduction in moderate to severe cancer pain patients. However, there was a trend of reduced opioid consumption. Further studies with larger sample sizes, longer duration, or higher doses of nefopam are warranted.

**Registration:** Thai Clinical Trail Registry (TCTR) ID TCTR20181016001; registered on 12 October 2018.

## Introduction

Pain is one of the most common and distressing cancer symptoms. Despite development of new drugs and modalities for treating cancer pain during the past decade, around half of patients with cancer suffer from moderate to severe pain, which impacts their quality of life ^1^ and may result in various psychosocial responses. The prevalence of pain among patients with cancer remains high worldwide. A systematic review and meta-analysis showed pooled pain prevalence rates of 39.3% after curative treatment, 55% during cancer treatment, 66.4% in advanced or terminal stages, and 50.7% in all cancer stages ^2^.

World Health Organization guidelines recommend starting medications such as non-steroidal anti-inflammatory drugs (NSAIDs), paracetamol, or opioids in all adults with cancer-related pain when pain management is initiated ^3^. However, these drugs may be contraindicated for some patients. Additionally, these medications may result in various intolerable adverse effects. For example: opioids can cause nausea, vomiting, constipation, drowsiness, and dry mouth ^4^; paracetamol may induce hepatotoxicity ^5^; and NSAIDs should be used with caution in high-risk patients who are aging or with pre-existing renal or gastrointestinal disease, hypovolemia, prior peptic ulcer disease, and renal impairment ^6^.

Nefopam is a centrally acting non-opioid, non-steroidal analgesic agent that inhibits the reuptake of serotonin, norepinephrine, and dopamine ^7^. It also inhibits calcium influx and blocks voltage-sensitive sodium channels, which leads to decreased activation of post-synaptic glutamatergic receptors such as N-methyl-D-aspartate (NMDA) receptors ^8^. Nefopam does not bind to the opioid receptors that may suppress respiration and has no anti-inflammatory or antiplatelet effects.

The role of nefopam in treating pain has been investigated in several animal models and clinical human studies. Nefopam was found to be an effective analgesic adjuvant, with studies showing some benefits in reducing postoperative opioid consumption following abdominal and orthopedic surgeries ^9, 10^. However, few studies have evaluated the efficacy of nefopam for cancer pain. It remains unclear whether nefopam has value as an adjuvant or alternative to analgesia in management of cancer pain. This study aimed to evaluate the analgesic efficacy of nefopam for moderate to severe cancer pain. We also evaluated concurrent morphine consumption and adverse effects during nefopam administration.

## Methods

### Study design

This double-blinded randomized controlled trial was approved by the Ramathibodi Hospital Ethical Committee (approval number, MURA2018/368) and was registered to the Thai Clinical Trials Registry (TCTR) on 12 October 2018 ( TCTR20181016001). Written informed consent was obtained from patients to participate in the study. This study was conducted among patients with cancer who were admitted to Ramathibodi Hospital between October 2018 and March 2019.

### Patients and randomization

Inclusion criteria were patients with cancer who: 1) were over 18 years of age, 2) had moderate to severe cancer pain defined by a numeric rating scale (NRS) score of 4/10 or over, and 3) required a strong opioid. Exclusion criteria were patients who were: 1) unable to report on the NRS, 2) unable to use the intravenous patient-controlled analgesia (IV-PCA) machine, 3) currently receiving radiotherapy or chemotherapy (within the past 3 weeks), 4) within 1 week postoperative, 5) receiving a monoamine oxidase inhibitor (within the past 30 days), 6) with a history of convulsive disorder, ischemic heart disease, or allergy to morphine, 7) pregnant, and 8) breastfeeding.

After eligible patients signed the informed consent form, they are blindly randomized into two groups (a nefopam group and placebo group) by stratified permuted block randomization in a 1:1 ratio base on two stratification factors: sex (male vs. female) and current opioid use (naïve opioid user vs. non-naive opioid user). The randomization was performed by a research assistance who was not involved in the study. Allocation of the patients to each study group was concealed in an opaque envelope. All participants and outcome assessors were blinded to the group allocation.

### Intervention

The nefopam group received 20 mg of nefopam in 100 ml normal saline (NSS) intravenously via an infusion pump over 30 minutes every 8 hours for a 24-hour period. The placebo group received 100 ml NSS intravenous infusion with a similar protocol. The agents, prepared by a nurse who was not involved in the study, were in identical appearance bottles; 100 ml of transparent, colorless solution, containing either nefopam (Acupan® BIOCODEX) or placebo. Other current medications were continued throughout the study period. IV-PCA with morphine was given for 48 hours using a similar protocol for all patients (PCA only with morphine 1 mg/ml, PCA dose 1 mg, lock-out interval of 5 minutes).

All patients were educated on rating their pain from 0–10 using the NRS and operating the IV-PCA machine. Morphine consumption, NRS, blood pressure, and heart rate were measured every 4 hours over a 48-hour period and reported side effects were also collected. Significant pain reduction was defined as a decrease on the NRS of at least 30% from the initial score. New onset of tachycardia was defined as a heart rate equal to or higher than 120 beats per minute (bpm) that had increased by 20% from baseline measurements.

The patients were withdrawn and labels were opened if they exhibited a heart rate over 150 bpm, arrhythmia, development of extreme unexpected events (e.g. pulmonary embolism, acute ischemic heart disease), or if the patient could not continue to use the PCA machine during the study period.

### Statistical analysis

With a desired power of 0.8, alpha level of 0.05, and a 30% dropout rate, the calculated sample size was 20 patients per group ^11^. Student’s t-tests or Mann-Whitney tests were used to test differences between groups for continuous variables such as body weight and cumulative morphine consumption. Fisher’s exact or chi-square tests were used for categorical variables such as gender and side effects. Repeated measures analysis of variance was used to compare blood pressure and heart rate between the groups over time. SPSS version 20.0 (IBM Corp. Released 2011, IBM SPSS Statistics for Windows, Armonk, NY) was used for all analyses. A *p-*value less than 0.05 was considered to indicate statistical significance.

## Results

**Figure 1.**
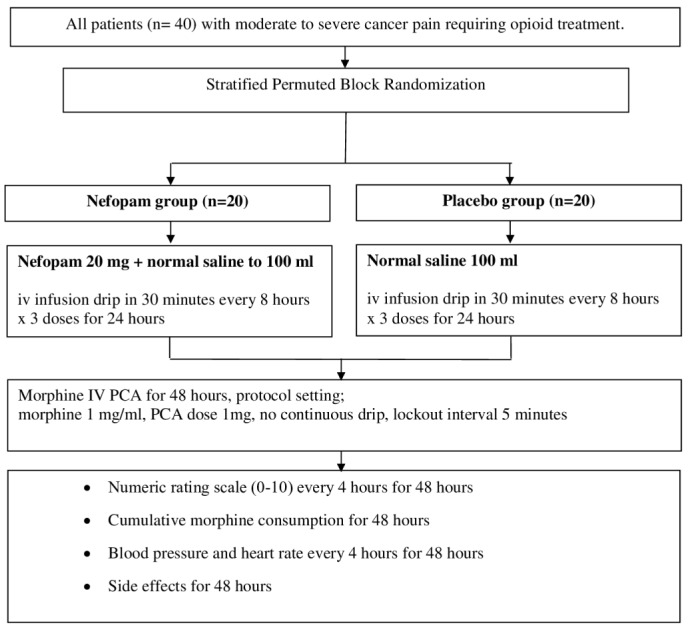
CONSORT study flow chart.

**Figure 2.**
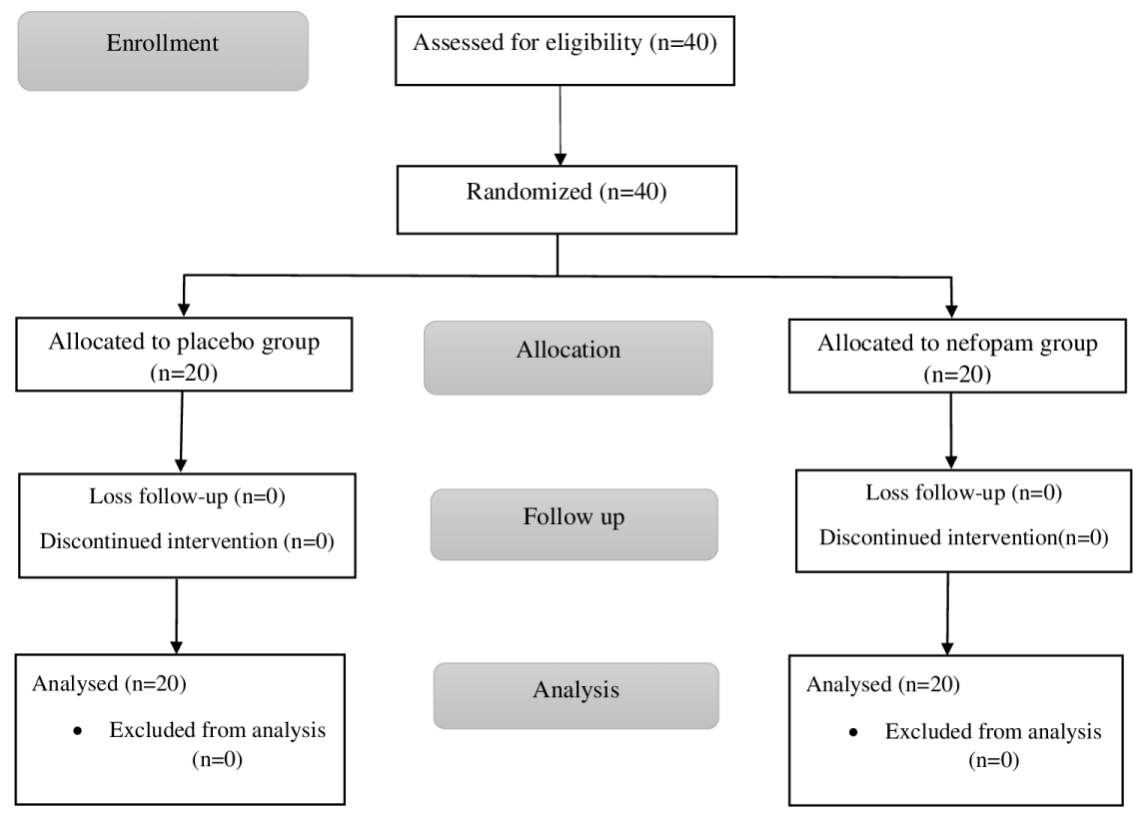
Flow diagram of patient enrollment.

In total, 40 patients were assessed for eligibility and randomized to a nefopam group or a placebo group (20 patients in each group). Figure 1 and Figure 2 show the study flow chart and patient enrollment, respectively.

The most common cancer type was gastrointestinal cancer (32.5%), and the majority of patients (67.5%) had metastases; consequently, the majority (67.5%) already had prescriptions for opioids for pain management at the time of enrollment. Some patients had also been prescribed analgesic adjuvants, such as tricyclic antidepressants and gabapentinoids. Demographic data for both groups are presented in Table 1. There was homogeneity across all parameters considered.

In the nefopam group, 65% of patients had significant pain reduction at 12 hours, 80% at 24 hours, 85% at 36 hours, and 65% at 48 hours. In the placebo group, there was significant pain reduction for 70% of patients at 12 hours, 75% at 24 hours, 80% at 36 hours, and 60% at 48 hours. There were no statistically significant differences between the groups at any measurement point (p>0.05; Table 2).

**Table 1.** Demographic data.

	Nefopam (n=20)	Placebo (n=20)	*p*-value
Sex (M/F)	10/10	8/12	0.525
Age (years)	54.35 ± 14.55	55.15 ± 12.51	0.853
Weight (kg)	53.25 ± 11.35	53.75 ± 7.98	0.873
Height (cm) Type of cancer Gastrointestinal Liver Head and neck Breast Urological Gynecological Lung Lymphoma	162.45 ± 10.61 7 1 3 2 3 2 1 1	159.15 ± 9.98 6 3 3 4 1 1 2 -	0.317 0.732
Opioid naïve patients /non-opioid naïve patients Patients with adjuvants/ patients without adjuvants Metastatic cancer (yes/no) Baseline pain intensity (NRS) Baseline pain severity mild moderate severe	8/12 6/14 12/8 7.05 ± 1.96 - 12 8	5/15 9/11 15/5 6.50 ± 1.61 - 15 5	0.311 0.327 0.311 0.443 0.311

Data presented as mean ± standard deviation or frequency. NRS, numeric rating scale.

**Table 2.** Effectiveness of nefopam for significant pain reduction **.

	Nefopam (n=20) n (%)	Placebo (n=20) n (%)	*p*-value
12 hours	13 (65)	14 (70)	0.736
24 hours	16 (80)	15 (75)	>0.999
36 hours	17 (85)	16 (80)	>0.999
48 hours	13 (65)	12 (60)	0.744

** Significant pain reduction defined as at least a 30% decrease on the numeric rating scale from the initial value. Chi-square or Fisher’s exact test.

**Figure 3.**
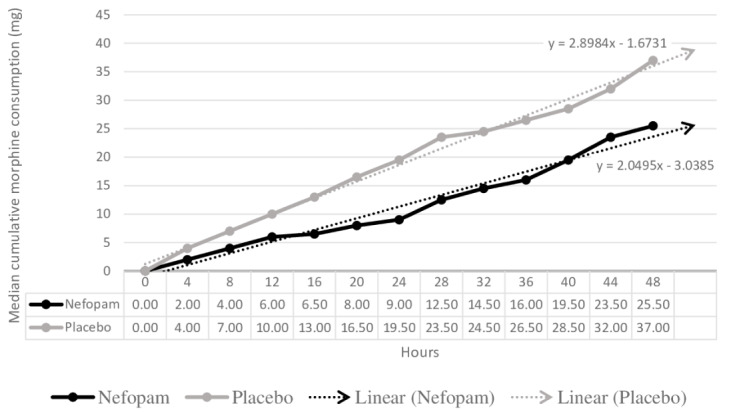
Effectiveness of nefopam for median cumulative morphine consumption.

The median cumulative dose of morphine consumption in 48 hours was lower in the nefopam group (25.5 mg) compared with the placebo group (37 mg), but there were no statistically significant differences at any measurement point ( Figure 3).

**Figure 4.**
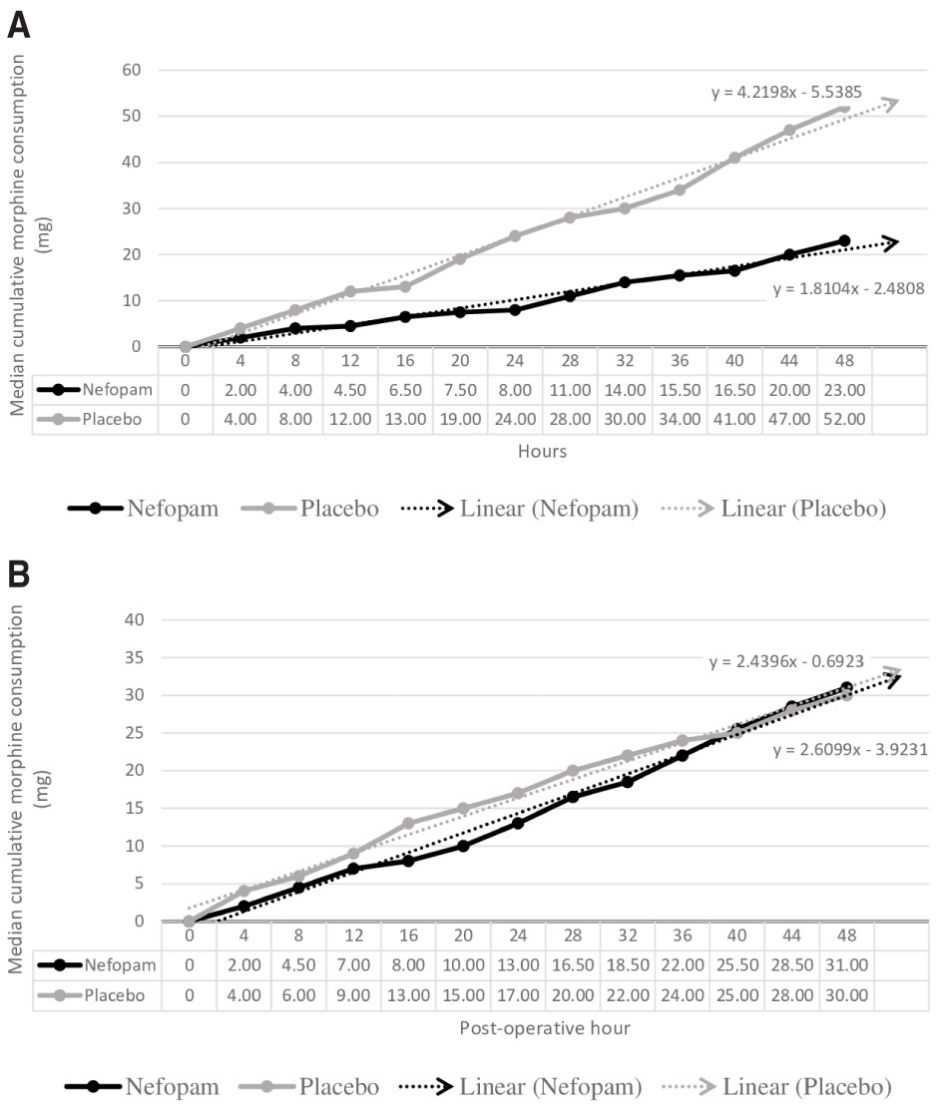
Effectiveness of nefopam for median cumulative morphine consumption (mg) by current opioid status. ( **A**) Opioid naïve subgroup; ( **B**) non-opioid naive subgroup.

Figure 4 shows the median cumulative dose of morphine in the opioid naive ( Figure 4a) and non-opioid naive subgroups ( Figure 4b). In the opioid naive subgroup, the median cumulative dose of morphine was lower in patients in the nefopam group at all measurement points, but this was not statistically significant.

**Figure 5.**
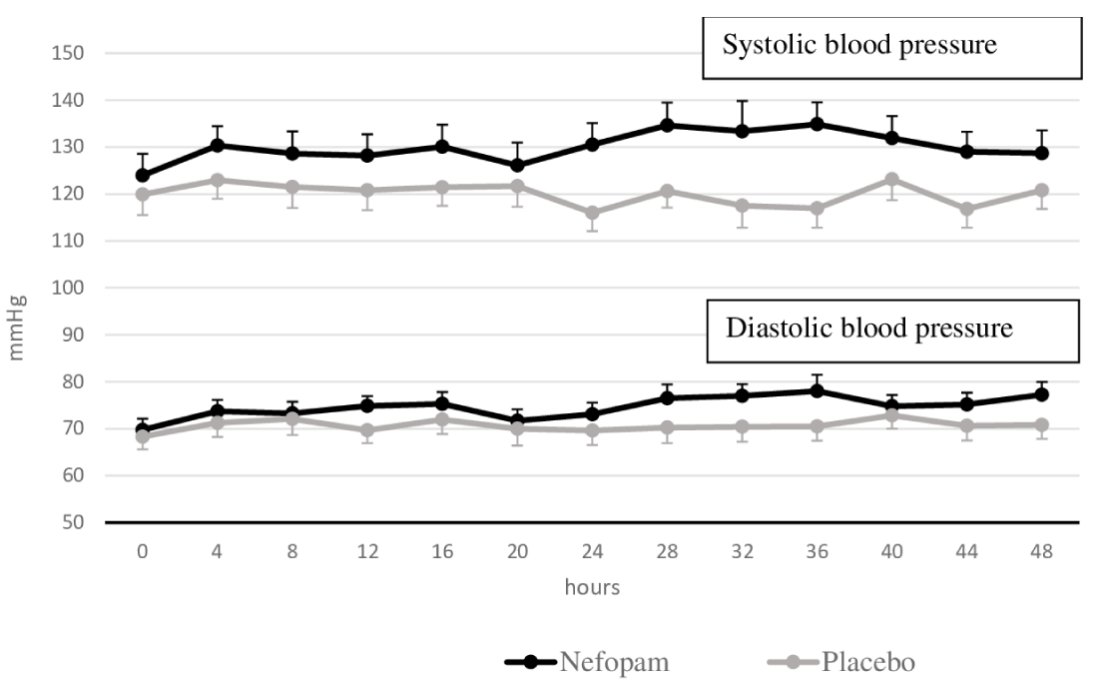
Blood pressure. Mean systolic/diastolic blood pressure and standard error of the mean for the two groups of patients.

**Figure 6.**
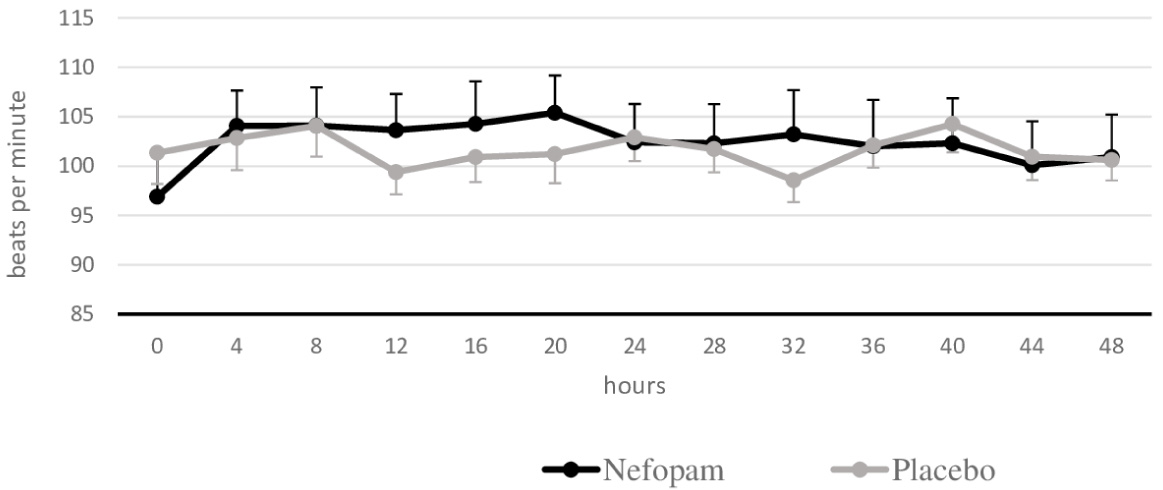
Heart rate. Mean heart rate and standard error of the mean for the two groups of patients.

Figure 5 shows the comparison of the mean systolic and diastolic blood pressure between the two groups. Figure 6 compares the mean heart rate between the two groups. There were no statistically significant differences in mean systolic blood pressure, mean diastolic blood pressure, or mean heart rate at all measurements ( *p*=0.066, *p*=0.213, and *p*=0.84, respectively). However, we found new onset of tachycardia in three patients (15%) in the nefopam group and four patients (20%) in the placebo group ( *p*=0.68).

Side effects such as nausea, vomiting, sweating, dry mouth, dizziness, and drowsiness were found in both groups ( Table 3). However, there were no statistically significant differences between the groups for any side effects.

**Table 3.** Side effects.

	Nefopam (n=20) n (%)	Placebo (n=20) n (%)	*p*-value
Nausea	6 (30)	5 (25)	0.723
Vomiting	2 (10)	1 (5)	>0.999
Sweating	5 (25)	5 (25)	>0.999
Dry mouth	8 (40)	5 (25)	0.311
Dizziness	3 (15)	1 (5)	0.605
Drowsiness	5 (25)	1 (5)	0.182

Chi-square or Fisher’s exact tests.

## Discussion

In published guidelines, most recommendations concerning nefopam have focused on acute and postoperative pain control ^8, 12– 15^. A quantitative systematic review drew three main conclusions for nefopam in postoperative pain prevention: 1) nefopam had a morphine-sparing effect, 2) it decreased pain intensity at 24 hours postoperatively, and 3) it increased the risk for tachycardia and sweating ^16^. Although the effectiveness of nefopam in chronic cancer- and non-cancer-related pain remains unclear, recent studies have investigated the analgesic protocols for nefopam, its effect on hyperalgesia, and its role in neuropathic pain ^8, 17, 18^.

Evidence for the use of nefopam for cancer pain remains limited. Minotti and coworkers ^19^ evaluated the analgesic efficacy of oral diclofenac, nefopam, and aspirin with codeine for chronic cancer pain, and found statistically significant pain relief for all treatments; however, that study had a high dropout rate (73.7%). In our study, the rescue of any breakthrough pain by IV-PCA was thought to prevent dropout because of insufficient pain control. In addition, the intravenous infusion protocol might have been able to prevent dropout because it decreased the risk for side effects.

The pathophysiology of cancer-related pain is complex and may include several mechanisms such as local and systemic inflammatory responses, direct tumor-related pain, metastatic cancer-induced bone pain, and neuropathic pain ^20– 23^. Central sensitization can also make cancer pain management more complicated. Its “wind up” phenomenon is also activated via NMDA receptors ^24^. Nefopam is a non-opioid analgesia that is considered to act centrally. Its main mechanisms of action involve the inhibition of serotonin, norepinephrine, and dopamine reuptake and modulation of calcium and sodium channels, which leads to decreased activation of postsynaptic glutamatergic receptors (e.g., NMDA receptors) that are involved in development of hyperalgesia ^8^. Consequently, we hypothesized that nefopam could be used as an adjuvant or alternative analgesia for cancer pain based on its various mechanisms of analgesic action. 

To the best of our knowledge, this study is the first randomized controlled study to evaluate efficacy of intravenous nefopam in moderate to severe cancer pain. However, nefopam did not show statistically significant pain reduction compared with placebo. This may be explained by various reasons. First, the drug potency might have been too low to control moderate to severe cancer pain. Some studies have suggested that in surgical settings, nefopam 20 mg should be equipotent to morphine 6–12 mg ^25^ or meperidine 50 mg ^26^. Its plasma half-life is 3–5 hours and plasma peak concentration is reached after 30 minutes of continuous intravenous infusion ^16^. Previous studies ^10, 27^ that evaluated higher doses of nefopam (maximum dose of 120 mg per day) in hepatic resection and orthopedic surgery found nefopam had a superior analgesic effect. We investigated the efficacy of nefopam at 60 mg per day, which had been found to be the effective dose 80 for moderate post-operative pain ^28^. However, this dose might have been too low for moderate to severe cancer pain. Second, the treatment period might have been too short to allow discrimination of any differences. Third, the complex mechanisms of chronic cancer pain would be considerably hindrances of effective pain reduction.

Although many previous studies ^9, 10, 28– 32^ on postoperative pain have shown that nefopam reduced morphine consumption at 24–48 hours for 30–50% of patients, our study did not confirm this finding. However, the median cumulative dose of morphine showed a lower trend in the nefopam group at all measurement points, especially in opioid naive patients receiving nefopam. Further studies focusing on nefopam in opioid naive patients with cancer that include a larger sample size, have a longer duration, or use higher doses of nefopam may be beneficial. 

We found that side effects such as nausea, vomiting, sweating, dry mouth, dizziness, tachycardia, and drowsiness were comparably low in both groups. In one quantitative review, the results showed an increased risk for tachycardia (21.3%) and sweating (8.8%) in postoperative patients receiving nefopam ^16^. However, the rate of side effects may be dissimilar in different patients and clinical settings. The low rate of adverse effects in this study might have been due to the mode of drug administration. Continuous intravenous administration avoids the peaks associated with periodic administration, as used in earlier study protocols.

There were some limitations in this study. We only investigated one dosage of intravenous nefopam (60 mg per day). This may be considered the lowest effective dose and, combined with the short duration of administration, was considered to reduce the risk for unfavorable adverse effects ^28^. Therefore, the low dose of nefopam and the short study period may explain why our study could not show differences in pain reduction between the two groups. Additionally, the baseline opioid dose in the non-opioid naive subgroup was not controlled, which might have affected the results. In addition, we did not evaluate pain characteristics, psychological parameters, and quality of life, which may have provided helpful information. Moreover, the incidence of analgesic-related side effects might have been underestimated because evaluation of side effects was only performed after 48 hours. Therefore, the incidence of temporary side effects that developed might have been omitted.

## Conclusion

At a dosage of 60 mg per day, nefopam did not provide significant pain reduction in patients with moderate to severe cancer pain. However, this study showed a trend towards reduction of opioid consumption in the nefopam group. Further studies with larger sample sizes, longer duration, or higher doses of nefopam are warranted to further investigate the efficacy of nefopam for cancer pain.

## Data availability

### Underlying data

Figshare: Data of nefopam in cancer pain, https://doi.org/10.6084/m9.figshare.12177423 ^33^.

### Reporting guidelines

Figshare: CONSORT 2010 Checklist for "Analgesic efficacy of nefopam for cancer pain: A randomized controlled study", https://doi.org/10.6084/m9.figshare.12249569 ^34^.

Data are available under the terms of the Creative Commons Attribution 4.0 International license (CC-BY 4.0).
