# Supplementary material for: Analgesic efficacy of nefopam for cancer pain: a randomized controlled study
Source: F1000Res. 2020 May 19;9:378. [Version 1] doi: 10.12688/f1000research.23455.1 (PMC7276938; doi:10.12688/f1000research.23455.1)
Supplement: Peer review history

# Peer review: Reviewer response for version 1

**DOI:** 10.5256/f1000research.25886.r63651

**Published:** 2020-06-04

**Authors:** Abdul Hadi Mohamed

**Affiliations:** 1 Department of Anaesthesiology and Critical Care, Kulliyyah of Medicine, International Islamic University Malaysia, Kuantan, Malaysia

This study is very relevant to our today practice and important for the patients’ well-being and continuous health-care after suffering from cancer pain.

 Overall coverage and discussion are appropriate.

 The main limitation for this study is the number of sample and monitoring of the results which may hinder the purpose of the study.

 Adequate and covering the area of research but some area may need sources of the references.

 The methodology and flow of the study was clearly organized inclusive the randomization performed.

 This study was well constructed which included intravenous administration and back up with the patient controlled administration of morphine to hinder potential pain during the study.

 The table and the analysis of the result are adequate.

 The coverage of the discussion and suggestion are adequate.

 I would agree that the result might be different if the samples more that 20 patients for each arm.

 However, if the monitoring interval be shorter for examples 4 hourly or 6 hourly then we might see the significant different results of the study with the similar sample.

 Even-though Nefopam have multiple mode of action but how much they covered nociceptive and neuropathic pathway were not covered in this study and discussion.

 Cancer pain can be prominent either nociceptive or neuropathic pain and sometimes both which  causing dilemma for palliative to manages.

 It is good if in this study or discussion to highlight more on the aspect of nefopam coverage either on nociceptive or neuropathic or both to certain degree.

Is the work clearly and accurately presented and does it cite the current literature?

Partly

If applicable, is the statistical analysis and its interpretation appropriate?

Yes

Are all the source data underlying the results available to ensure full reproducibility?

Yes

Is the study design appropriate and is the work technically sound?

Yes

Are the conclusions drawn adequately supported by the results?

Yes

Are sufficient details of methods and analysis provided to allow replication by others?

Yes

Reviewer Expertise:

Pain and palliative

I confirm that I have read this submission and believe that I have an appropriate level of expertise to confirm that it is of an acceptable scientific standard.

# Peer review: Reviewer response for version 1

**DOI:** 10.5256/f1000research.25886.r63652

**Published:** 2020-06-01

**Authors:** Kok Yuen Ho

**Affiliations:** 1 The Pain Clinic, Mount Alvernia Hospital, Singapore, Singapore

This is a placebo-controlled double-blind RCT comparing nefopam and placebo in moderate to severe cancer pain. The methodology and analysis is properly done and the results are clear. Unfortunately, the sample size is small with only 20 patients per group. 

 As the patients in both groups were given an IV-PCA pump to use, I am not surprised that there was no difference in the NRS pain scores for 48 hours. A better indication would be a difference in the morphine consumption - which could have been the primary outcome of this study.

Is the work clearly and accurately presented and does it cite the current literature?

Yes

If applicable, is the statistical analysis and its interpretation appropriate?

Yes

Are all the source data underlying the results available to ensure full reproducibility?

Yes

Is the study design appropriate and is the work technically sound?

Yes

Are the conclusions drawn adequately supported by the results?

Yes

Are sufficient details of methods and analysis provided to allow replication by others?

Yes

Reviewer Expertise:

Pain Management

I confirm that I have read this submission and believe that I have an appropriate level of expertise to confirm that it is of an acceptable scientific standard.
